# Autonomic dysfunction in neurological disorders

**DOI:** 10.18632/aging.101896

**Published:** 2019-04-09

**Authors:** Li Xiong, Thomas W. H. Leung

**Affiliations:** 1 Division of Neurology, Department of Medicine and Therapeutics, The Chinese University of Hong Kong, Hong Kong SAR, China; 2 BrainNow Research Institute, Hong Kong Science and Technology Park, Hong Kong SAR, China

**Keywords:** autonomic dysfunction, neurological disorders

Autonomic dysfunction results in a diverse constellation of symptoms that can include every organ system. It accompanies not only the primary autonomic nervous system degenerations but is also responsible for much of the morbidity associated with frequently encountered neurological disorders, such as cerebrovascular disease, movement disorders, dementia, multiple sclerosis (MS) and peripheral neuropathies. There is a need to identify symptomatic dysautonomia in order to ensure appropriate management and reduce risk of increased morbidity and mortality in these individuals. In the early 1980, Ewing et al. proposed five simple non-invasive cardiovascular reflex tests which helped to diagnose autonomic dysfunction successfully. These are heart rate response to Valsalva maneuver, heart rate response to deep breathing and heart rate response to standing for assessment of parasympathetic functions and systolic blood pressure response to standing and diastolic blood pressure response to sustained handgrip for sympathetic functions [1]. The advantage of using such a battery is to allow classification of autonomic dysfunction according to severity in which autonomic function can be categorized into five groups (normal, early, definite, severe and atypical) depending on the number of abnormal or borderline results. Ewing’s battery of autonomic function tests has been widely used for peripheral autonomic disorders. In the past ten years, we observed it can also be used for central autonomic failures [2–4].

By using Ewing battery of autonomic function tests, our previous studies found autonomic dysfunction was frequently present in acute ischemic stroke with a prevalence of 76.0% [2,3], which may persist up to six-months after stroke onset [2]. This may result from damage of the cortical or subcortical structures or of the neural pathways known to regulate the autonomic system. Insular cortex lying within the middle cerebral artery territory is the most important cortical area controlling both sympathetically and parasympathetically mediated cardiovascular regulation. However, insular lesion was excluded in our protocols, it therefore seems that extrainsular lesions affect extrainsular central autonomic areas or their interconnecting fibers and thereby provoke cardiovascular autonomic activation. We further investigated autonomic dysfunction in post-acute ischemic stroke according to presumed etiology classified as large-artery atherosclerosis or small-vessel occlusion, and found that parasympathetic and sympathetic cardiovascular modulations were more severely impaired in patients with large-artery atherosclerosis [4]. Therefore, stroke etiology itself might play a pivotal role in autonomic dysfunctions in post-acute ischemic stroke. Recently, our prospective observational study demonstrated that autonomic dysfunction gauged by Ewing battery predicts poor functional outcome in 3 months after acute ischemic stroke [3]. All of these findings substantiated the importance of an early diagnosis of autonomic dysfunction in stroke survivors given the elevated risk of cardiovascular events and mortality.

Besides, degenerative or primary dysautonomia has been reported to be associated with hypokinetic movement disorders such as multiple system atrophy (MSA) and idiopathic Parkinson’s disease (PD), which are the main movement disorders associated with autonomic dysfunction [5]. There is a different pathological basis of autonomic dysfunction in MSA and PD, which plays an important role in the differential diagnosis of parkinsonian disorders with implications for response to treatment and prognosis. Allen et al. first investigated the autonomic dysfunction comparing the most common dementia subtypes in the elderly with Ewing battery: Alzheimer’s disease (AD), vascular dementia (VAD), dementia with Lewy bodies (DLB) and Parkinson’s disease dementia (PDD) [6]. PDD and DLB had considerable dysfunction. VAD showed limited evidence of autonomic dysfunction, whereas in AD, apart from orthostatic hypotension, autonomic functions were relatively unimpaired. To the best of our knowledge, all the common dementias have been associated with underactivity of the cholinergic nervous systems, and a generalized deficit in cholinergic function would be expected to lead to autonomic dysfunction. MS is a demyelinating disorder of the central nervous system that can result in varied neurological deficits, including motor dysfunction, sensory loss, bladder abnormalities and significant fatigue. Autonomic dysfunction caused by MS is attributed to multiple demyelinating plaques localized in the brain stem and spinal cord. However, there is not a consensus regarding the presence of the relationship between autonomic dysfunction and severity of MS, type of MS and expanded disability status scale.

In summary, autonomic dysfunction is commonly observed in neurological disorders. The purpose of this special issue elucidates the importance of an early diagnosis of autonomic dysfunction in patients with neurological disorders given the elevated risk of cardiovascular events and mortality. Currently, the aggressive medical treatment mainly focuses on those aspects of dysautonomia, most frequently encountered by the clinical neurologist such as orthostatic hypotension, bladder dysfunction and the disorders of gastrointestinal motility. Non-pharmacological treatment such as external counterpulsation, which is a noninvasive and well-established method to improve perfusion of vital organs, may be able to improve the impairment of autonomic function by decreasing the beat-to-beat blood pressure variability and increasing beat-to-beat heart rate variability [7,8]. Surgical procedure, described as transvascular autonomic modulation, utilizes an endovascular approach to dilate the thoracic venous system, resulting in mechanical stretching of the autonomic nerves and ‘resetting’ of the autonomic nervous system. However, there are no data at all to support the clinical utility of transvascular autonomic modulation and there is no scientific rational for the procedure. Therefore, we strongly encourage the development of novel approaches and therapeutic interventions for dysautonomia, but only when they are based on scientific rationale and supported by evidence of both safety and efficacy.

## References

[1] Ewing DJ, Clarke BF. Br Med J (Clin Res Ed). 1982; 285:916–18. 10.1136/bmj.285.6346.916 6811067 PMC1500018

[2] Xiong L, et al. Int J Stroke. 2013; 8:645–51. 10.1111/j.1747-4949.2012.00829.x 22759410

[3] Xiong L, et al. Stroke. 2018; 49:215–18. 10.1161/STROKEAHA.117.019312 29203690

[4] Xiong L, et al. J Neurol Sci. 2014; 337:141–46. 10.1016/j.jns.2013.11.036 24326200

[5] Shy GM, Drager GA. Arch Neurol. 1960; 2:511–27. 10.1001/archneur.1960.03840110025004 14446364

[6] Allan LM, et al. J Neurol Neurosurg Psychiatry. 2007; 78:671–77. 10.1136/jnnp.2006.102343 17178816 PMC2117678

[7] Xiong L, et al. J Stroke Cerebrovasc. 2017; 26:1487–92. 10.1016/j.jstrokecerebrovasdis.2017.03.007 28396189

[8] Tian G, et al. J Clin Neurol. 2016; 12:308–15. 10.3988/jcn.2016.12.3.308 27095525 PMC4960215

